# Potential of FX06 to prevent disease progression in hospitalized non-intubated COVID-19 patients — the randomized, EU-wide, placebo-controlled, phase II study design of IXION

**DOI:** 10.1186/s13063-022-06609-x

**Published:** 2022-08-19

**Authors:** Jan Kloka, Benjamin Friedrichson, Stephanie Dauth, Ann Christina Foldenauer, Anita Bulczak-Schadendorf, Maria J. G. T. Vehreschild, Francisco Maio Matos, Antoni Riera-Mestre, Antoinette D. I. van Asselt, Edoardo De Robertis, Vilma Traskaite Juskeviciene, Patrick Meybohm, Dana Tomescu, Karine Lacombe, Coen D. A. Stehouwer, Kai Zacharowski, on behalf of the IXION Collaboration Group, Oliver Old, Markus Ketomaeki, Lea Grebe, Patrick Booms, Simone Lindau, Sebastian Zinn, Isabel Maushagen, Timo Wolf, Christoph Stephan, Cathy Weynants, Sylvia Daamen, Petra Wülfroth, Thomas Steiner, Marinus van Hulst, Peter Kranke, Sebastian Hottenrott, Tobias Schlesinger, Benedikt Schmid, Daniel Röder, Eva Kranke, Tobias Haas, Philipp Schlesinger, Magdalena Sitter, Davide Valeri, Raquel Torres Iglesias, José María Mora-Luján, Adriana Iriarte, Pau Cerdà, Neringa Vaguliene, Andrius Macas, Jolanta Litviniene, Kristina Balne, Catarina Monteiro, Inês Antunes Ferreira, Patrícia Couceiro, Pedro Soares, Sofia Beirão, Yasmine Abi Aad, Thibault Chiarabini, Mihai Popescu, C. J. H. van der Kallen

**Affiliations:** 1 Department of Anaesthesiology, Intensive Care Medicine and Pain Therapy, University Hospital Frankfurt, Goethe University Frankfurt, Theodor-Stern Kai 7, 60590 Frankfurt, Germany; 2 Fraunhofer-Institute for Translational Medicine and Pharmacology ITMP, Frankfurt, Germany; 3 Fraunhofer Cluster of Excellence Immune-Mediated Diseases CIMD, Frankfurt, Germany; 4 Department of Internal Medicine, Infectious Diseases, University Hospital Frankfurt, Goethe University Frankfurt, Frankfurt am Main, Germany; 5 Centro Hospitalar e Universitário de Coimbra, E.P.E, Coimbra, Portugal; 6 Internal Medicine Department, Hospital Universitari Bellvitge, Barcelona, Spain; 7 Bellvitge Biomedical Research Institute (IDIBELL), Barcelona, Spain; 8 Faculty of Medicine and Health Sciences, Universitat de Barcelona, Barcelona, Spain; 9 Department of Epidemiology & Department of Health Sciences, University of Groningen, University Medical Center Groningen, Groningen, The Netherlands; 10 Department of Medicine and Surgery, University of Perugia, Perugia, Italy; 11 Department of Anaesthesiology, Lithuanian University of Health Sciences, Hospital of Lithuanian University of Health Sciences Kaunas Clinics, Kaunas, Lithuania; 12 Department of Anaesthesiology, Intensive Care, Emergency and Pain Medicine, University Hospital Wuerzburg, Würzburg, Germany; 13 “Carol Davila” University of Medicine and Pharmacy, Department of Anaesthesia and Intensive Care - Fundeni Clinical Institute, Bucharest, Romania; 14 Sorbonne Université, IPLESP, Hôpital St Antoine, AP-HP, Paris, France; 15 Department of Internal Medicine, Maastricht University Medical Centre, Maastricht, The Netherlands; 16 Cardiovascular Research Institute Maastricht (CARIM), Maastricht University, Maastricht, The Netherlands

**Keywords:** FX06, COVID-19, Disease progression, Capillary leak, Inflammation

## Abstract

**Background:**

More than 2.7 million hospitalizations of COVID-19-infected patients have occurred in Europe alone since the outbreak of the coronavirus in 2020. Interventions against SARS-CoV-2 are still in high need to prevent admissions to ICUs worldwide. FX06, a naturally occurring peptide in humans and other mammals, has the potential to reduce capillary leak by improving endothelial dysfunction and thus preventing the deterioration of patients. With IXION, we want to investigate the potential of FX06 to prevent disease progression in hospitalized, non-intubated COVID-19 patients.

**Methods:**

IXION is an EU-wide, multicentre, placebo-controlled, double-blinded, parallel, randomized (2:1) phase II clinical study. Patient recruitment will start in September 2022 (to Q2/2023) in Germany, Italy, Lithuania, Spain, Romania, Portugal, and France. A total of 306 hospitalized patients (≥ 18 years and < 75 years) with a positive SARS-CoV-2 PCR test and a COVID-19 severity of 4–6 according to the WHO scale will be enrolled. After randomization to FX06 or placebo, patients will be assessed until day 28 (and followed up until day 60). FX06 (2 × 200 mg per day) or placebo will be administered intravenously for 5 consecutive days. The primary endpoint is to demonstrate a difference in the proportion of patients with progressed/worsened disease state in patients receiving FX06 compared to patients receiving placebo. Secondary endpoints are lung function, oxygen saturation and breathing rate, systemic inflammation, survival, capillary refill time, duration of hospital stay, and drug accountability.

**Discussion:**

With IXION, the multidisciplinary consortium aims to deliver a new therapy in addition to standard care against SARS-CoV-2 for the clinical management of COVID-19 during mild and moderate stages. Potential limitations might refer to a lack of recruiting and drop-out due to various possible protocol violations. While we controlled for drop-outs in the same size estimation, recruitment problems may be subject to external problems difficult to control for.

**Trial registration:**

EudraCT 2021-005059-35. Registered on 12 December 2021. Study Code TMP-2204-2021-47.

**Supplementary Information:**

The online version contains supplementary material available at 10.1186/s13063-022-06609-x.

## Background

### Setting

SARS-CoV-2, officially referred to as severe acute respiratory syndrome-coronavirus-2 (SARS-CoV-2), has created an unknown challenge for healthcare systems worldwide. The virus was rapidly transmitted across geographic regions and triggered a pandemic due to the high infectivity, the ability to be spread even during the period of asymptomatic disease, and comparatively low virulence [1]. On December 8, 2019, the first case of the coronavirus disease 2019 (COVID-19) presented itself in the Hubei province of China [2]. Since then, the infection has spread worldwide, with nearly 433 million confirmed cases and 6 million deaths (World Health Organization situation report from March 8, 2022) [3].

The predominant clinical symptoms of COVID-19 are respiratory involvement, ranging from mild flulike illness to potentially fatal acute respiratory distress syndrome or fulminant pneumonia [1]. SARS-CoV-2 shares similar features with other coronaviruses, including the spherical morphology with spike projections on the surface as well as a high sequence identity, e.g. with SARS-CoV and SARS-like coronavirus (SL-CoV) [4]. In contrast to SARS-CoV, SARS-CoV-2 is less pathogenic but has a higher human-to-human transmissibility [5].

After SARS-CoV-2 infection, severe alveolar damage has been observed, which might be due to alveolar cells of the lung (type II) being more apt to be infected by SARS-CoV-2 [6]. SARS-CoV-2 infects host cells via angiotensin-converting enzyme 2 (ACE2) as its cellular receptor. This membrane-bound aminopeptidase receptor is predominantly expressed in humans in various tissues such as the heart, intestine, kidney, pulmonary alveolar cells, and ubiquitously on endothelial cells [7]. ACE2 counteracts the activity of angiotensin II and protects against harmful activation of the ACE system. It has been speculated that ACE inhibitors and angiotensin-receptor blockers (ARBs) may enhance ACE2 expression, thus predisposing to more severely COVID-19 progression [8]. SARS-CoV-2 has been shown to be capable of direct entry into engineered human blood vessel organoids in vitro [9]. The systemic impairment of microcirculatory function in various vascular beds might be explained by SARS-CoV-2-induced endotheliitis, which might also contribute to the clinically disastrous course of patients with severe COVID-19 [10].

The medication administered to patients, especially those with moderate to severe COVID-19, depends on the underlying pathologic features and the different clinical phases of the disease. Antiviral drugs (e.g. remdesivir), antiinflammation agents (e.g. dexamethasone), plasma, and hyperimmune immunoglobulins are part of the therapeutic options against COVID-19 [11]. Additionally, an emergency use authorization for combinational use of bamlanivimab and etesevimab for adult and pediatric SARS-CoV-2-positive patients with mild to moderate COVID-19, who are at risk of developing severe COVID-19, was granted by the FDA [12]. Approved uses include treatment of individuals aged 65 years or older and/or suffering from certain chronic diseases. Treatment with bamlanivimab and etesevimab is not authorized for patients hospitalized or receiving oxygen in context of a SARS-CoV2 infection, because the respective data are not available. Moreover, the delivery of monoclonal antibodies such as bamlanivimab and etesevimab to hospitalized COVID-19 patients in need of high flow oxygen or mechanical ventilation may be related to worse clinical outcomes [12].

This offers a justification for treatments stabilizing the endothelium with anti-inflammatory drugs such as anti-cytokine directed drugs like tocilizumab. A severe cytokine storm as caused by COVID-19 is followed by oedema, capillary leakage syndrome, and consequently dysfunction of the lungs and other organs [13].

There is still a lack of therapeutical options and a high medical need for efficacious and safe therapies for patients with COVID-19.

FX06, a naturally occurring peptide, might be an innovative promising strategy to improve endothelial dysfunction, capillary leak, arterial oxygenation, and lung function and possibly be able to prevent disease progression.

In a case report, FX06 was used to reduce capillary leak syndrome and to improve lung function in a patient with Ebola infection. FX06 administration led to a substantial improvement of the vascular leak syndrome and respiratory parameters. Additionally, the detection of Ebola-virus-specific antibodies was accompanied by a decrease in viral load [14].

In two German tertiary care university hospitals, six COVID-19 patients who suffered from moderate to severe ARDS at ICU admission and were mechanically ventilated were treated with i.v. FX06 (400–600 mg per day; 3–7 days) as part of an elective rescue treatment of seriously ill COVID-19 patients [15]. Patients were between 51 and 78 years of age; all had at least one comorbidity (e.g. obesity, type 2 diabetes mellitus, bronchial asthma) and all patients needed invasive ventilation. In the course of their disease, five out of these six patients required additional ECMO treatment. In the first 3 days after the start of FX06 administration, the mean oxygenation ratio improved, returned to baseline, and then increased steadily thereafter from the seventh day.

In a phase II study, 234 patients with acute myocardial infarction received FX06 in a dosage of 400 mg intravenously separated into two bolus injections in a 10-min interval. The therapy with FX06 showed a significant reduction of the necrosis zone 5 days after the event, evaluated by MRI. Patients treated with FX06 also showed a trend towards improvement of a combined clinical endpoint of mortality and any cardiac event. Numerically less serious cardiac events occurred in the FX06-treated group than in the placebo group, and there were no differences in adverse events [16].

FX06 might be an innovative promising strategy to improve endothelial dysfunction, capillary leak, arterial oxygenation, and lung function possibly able to prevent disease progression.

## Methods

The study was planned in concordance with the SPIRIT statement guideline to ensure complete reporting of the study [17]. Study protocol V1.1 (EudraCT no. 2021-005059-35), patient information, and informed consent were approved by the Ethics Committee of the University of Frankfurt, Germany (Ref: 2021-512-AMG, Chair: Prof. Dr. Harder) and of all participating centres on 18 February 2022. At the same time, the study documents have been approved by the responsible federal authority (Bundesinstitut für Arzneimittel und Medizinprodukte – No. 61-3910-4045190) according to the requirements of §42 Abs. 2 AMG and §9 GCP-V. Each patient must give written informed consent to participate in the study.

Protocol modifications to ongoing studies must be made via amendment and they will be communicated via the EU. The sponsor is responsible to obtain independent approval for the amendment from the federal regulatory authority and a positive opinion from the competent ethics committees if required according to GCP. If applicable, regulatory authority must be notified about the amendment. The sponsor will prepare a complete, integrated final clinical study report of the study. This report will be signed by the coordinating investigator of the study. The investigator/investigator(s) will each receive a copy of this report.

Publication(s) will be prepared according to standard guidelines, authorships are determined internally and according to authorship guidelines (e.g. Good Publication Practice and Recommendations for the Conduct, Reporting, Editing, and Publication of Scholarly Work in Medical Journals).

### Trial design

**Fig. 1:**
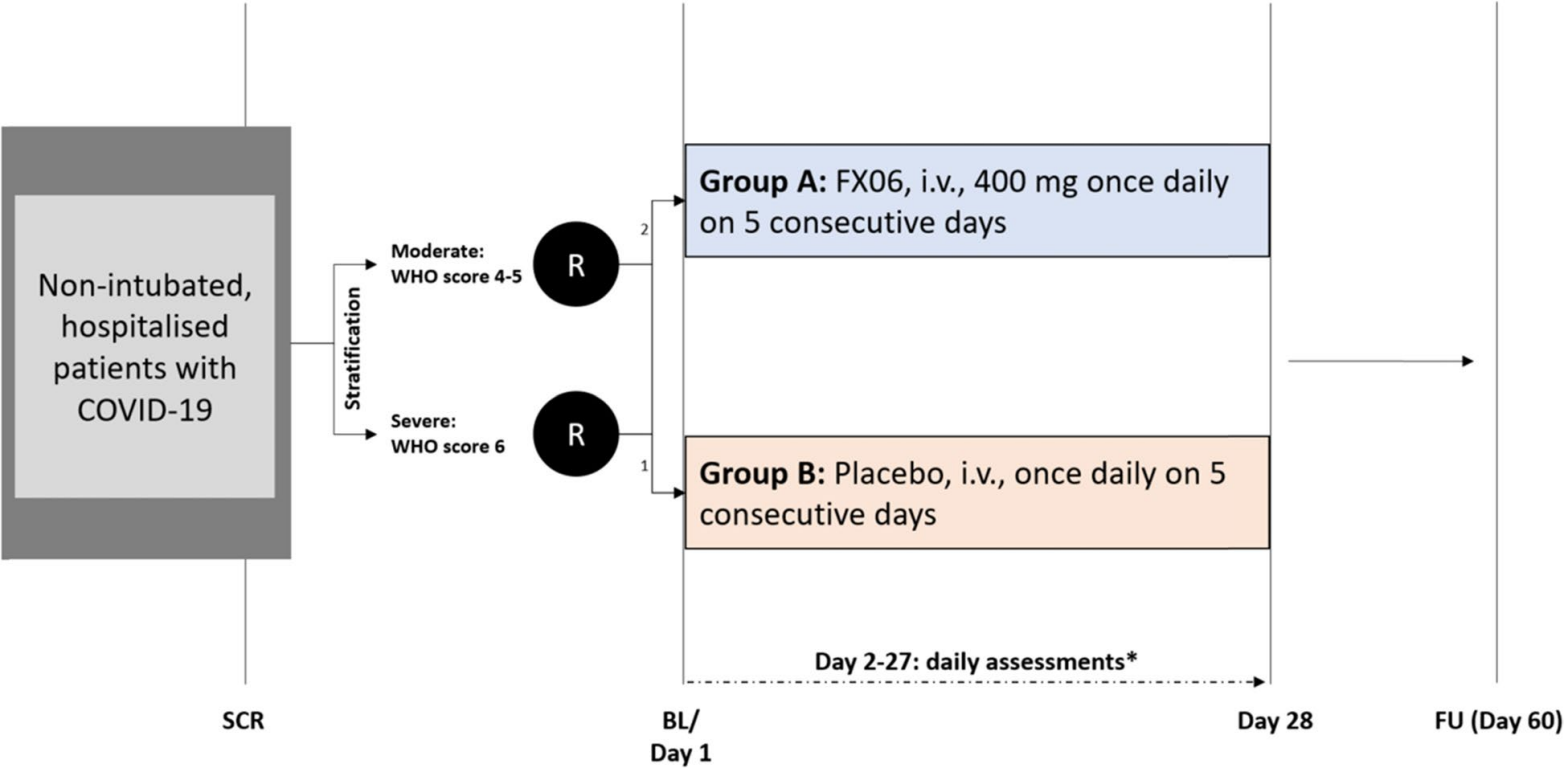
Study design of IXION. Hospitalized, non-intubated COVID-19 patients will be included in this study and stratified by WHO score. Patients will then be randomized 2:1 to either FX06 or placebo. FX06 or placebo will be administered intravenously for 5 consecutive days. Patients will be assessed and observed for 28 days and followed up until day 60. SCR screening, BL baseline, FU follow-up, i.v. intravenous, R randomization, WHO World Health Organization. *Day 2–27 will include personal visits as well as remote “visits”

IXION will be an EU-wide, multinational, placebo-controlled, double-blinded, parallel, randomized (2:1), phase II clinical study to investigate the superiority of FX06 compared to standard of care to prevent disease progression in hospitalized non-intubated COVID-19 patients. In total, 306 patients fulfilling inclusion criteria will be stratified according to their WHO severity group (moderate: score 4–5 or severe: score 6) and by centre and randomized 2:1 to either one of the two treatment groups (FX06 or placebo) (Fig. 1).

**Table 1:**
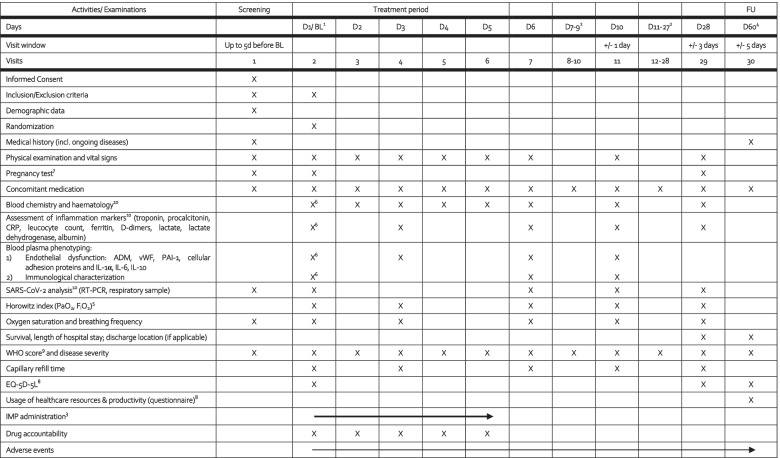
Timeline of IXION. Overview of assessments and time points of visits during the study period

^1^Screening and baseline should be maximally 5 days apart, but can also be performed at the same day; ^2^if patients are not in the hospital anymore, patients can be contacted remotely to retrieve the information; ^3^IMP administration will be performed at BL and the following 4 days; ^4^FU can be performed as a telephone call and will be performed at day 60; ^5^lung function parameters will only be documented if they are routinely measured; ^6^blood sample collection before first dose of IMP; ^7^only in women of childbearing potential; urine pregnancy test; ^8^to be administered as interview; ^9^WHO score will be eligible for the whole study period for evaluation of disease severity also if patients become SARS-CoV-2 negative during the study period; ^10^will be done in clinical routine by the local laboratory and will be documented for the study

Due to the multinational design, patients will be recruited in Germany, Italy, Lithuania, Spain, Romania, Portugal, and France.

### Participants

In this study, we will investigate the potential of FX06 to prevent COVID-19 progression in hospitalized, non-intubated COVID-19 patients. We will also investigate the influence on disease improvement, lung function, endothelial dysfunction, capillary leak, and arterial oxygenation in SARS-CoV-2 positive, hospitalized patients. Therefore, in this study, hospitalized, non-intubated COVID-19 patients will receive FX06 (in addition to standard of care (SoC)) or placebo (in addition to SoC) over 5 days. Patients will be observed until day 28 (and followed up at day 60) (Table 1).

Patients and potential study participants are identified by the study centres of the clinical partners and included in the study upon enrolment according to the following criteria:

### Inclusion criteria

- SARS-CoV-2 infection confirmed by PCR test
- Hospitalized patients
- WHO scores 4–6
- Oxygen saturation ≤ 92% under room air
- Breathing frequency per minute ≥ 20
- Patients ≥ 18 years and < 75 years
- Written informed consent obtained prior to the initiation of any protocol-required procedures by the patient
- Willingness to comply to study procedures and study protocol
- Patients able to understand the requirements of the study and give written informed consent

### Exclusion criteria

- Significant underlying known comorbidities or conditions, defined as:
  - Other severe advanced or chronic lung diseases (e.g. COPD Gold ≥ III, severe silicosis)
  - End-stage chronic kidney disease (stage 5)
  - End-stage chronic heart failure (NYHA ≥ III)
  - Dementia
  - Baseline neurologic disease which would preclude rehabilitation potential
  - Disseminated and/or metastasized malignancy
  - Severe deconditioning with a life expectancy of less than 6 months according to the treating physician
  - Immunocompromized patients:
    I. Recipient of a solid organ transplant
    II. Regular intake of anti-inflammatory therapy due to concomitant auto-immune disease (e.g. biologics)
    III. Primary immune deficiency
- Evidence of other significant uncontrolled concomitant diseases or serious and/or uncontrolled diseases with a bad prognosis that are likely to interfere with the evaluation of the patient’s safety and with the study outcome as judged by the treating physician
- Women pregnant or breastfeeding
- Males or females of reproductive potential not willing to use effective contraception for the duration of the study period
- Current participation in another interventional clinical trial with IMP or participation within the last 30 days

### Intervention trial drug — FX06

FX06 is a naturally occurring peptide in humans and mammals, Bβ15-42, derived from the E1 fragment of fibrin. The mechanism of action of FX06 is an important new discovery in understanding acute inflammation and oedema formation. FX06 competes with E1 fragments of fibrin for binding to an endothelial specific molecule, VE-cadherin, thereby acting as an anti-inflammatory, and it signals through VE-cadherin, thereby reducing plasma leakage into tissues [16, 18]. Based on animal models of vascular leakage and systemic inflammation, FX06 has considerable therapeutic potential for all diseases and pathological conditions associated with increased vascular permeability [19, 20]. FX06 binds to vascular endothelial (VE)-cadherin, preventing VE-cadherin-dependent transmigration of leukocytes [21]. Besides many different animal models of shock (septic, haemorrhagic, hypovolemic), FX06 was also tested in acute and chronic models of myocardial ischaemia and reperfusion injury in rats, mice and pigs [18] [20, 22]. FX06 reduced relative infarct size by more than 40%; this effect size is comparable to ischaemic preconditioning. The peptide is synthetically produced for exogenous human administration [21]. FX06 was well tolerated in the phase I study [21].

FX06 has been evaluated in a phase IIa clinical trial of first-time acute STEMI (ST-segment elevation myocardial infarction) patients undergoing primary percutaneous coronary intervention. In this proof-of-concept trial, FX06 reduced the necrotic core zone as one measure of infarct size on magnetic resonance imaging and appeared safe and well tolerated.

Furthermore, FX06 has been applied in a severe case of an Ebola virus-infected patient under compassionate use. The patient recovered after critical illness and no drug-related safety concerns were raised [21].

An investigator-initiated trial (IIT) in France treated mechanically ventilated patients with critical COVID-19 in a phase IIa study using the investigational product FX06/placebo (EudraCT no. 2020-002056-20).

Since there were no safety concerns in the phase II IIT study with FX06 in France/Paris, in which FX06 was administered intravenously for 5 days (2 × 200 mg per day), it was therefore decided to use the same dosage regimen.

### Randomization

A randomization list will be generated by an authorized person through the sponsor. Central randomization will be performed in a 2:1 allocation to FX06 and placebo stratified by WHO group (moderate: score 4–5 and severe: score 6) and centre. As randomization procedure, stratified block randomization will be applied. The randomization will be performed centrally within the eCRF System Open Clinica. Independent pharmacists will dispense the allocated treatment according to the computer-generated randomization list.

### Allocation

The randomization list is not available to blinded study staff including treating physicians. Within the clinics, treatment allocation will be concealed by providing placebo IMP for FX06, which will be of identical appearance in comparison to the study drug.

### Implementation

The randomization sequence is generated and validated prior to study start by the data management. Eligible patients are enrolled by the treating investigator who allocates the patients to the next randomized treatment according to the computer-generated randomization list. Blinded IMP is then given according to randomization number.

### Blinding

The study is randomized and blinded to subjects, therapists, and assessors (double-blinded). Treatment allocation remains concealed throughout the study, minimizing the risk of assessment and selection bias and assessment bias.

The treatment allocation also remains blinded for the study statistician until the final analysis to avoid early data-driven conclusions.

### Study objectives

#### Primary objective and endpoint

The primary objective is to demonstrate a difference in the proportion of patients with progressed/worsened disease state in patients receiving FX06 compared to patients receiving placebo until day 28.

- Proportion of patients with worsened disease state until day 28 in both treatment groups

Progression/worsening of disease state will be defined as a worsened WHO score on 3 consecutive days (always compared to the BL WHO score) until day 28 (e.g. a WHO score of 5 on 3 consecutive days for a patient that had a WHO score of 4 at BL will be defined as a worsened disease)

The assessment of disease severity and progression by the WHO score was chosen based on the recommendation of the WHO [23] to include this score for all COVID-19 clinical trials. Furthermore, for this international, multicentre study, it facilitates comparable and objective assessment of disease severity across study centres.

#### Secondary objectives and endpoints

Assessment and treatment comparison of the following objectives at all available visits and time points between the FX06 and placebo group:

- Disease progression/improvement (WHO scale score)
  - Proportion of patients with improved or progressed disease state
  - Proportion of patients with each WHO score
  - Average change in WHO score compared to BL
  - Average WHO score
  - Proportion of patients in each disease severity state (uninfected, mild, moderate, severe, dead)
  - Proportion of patients who need mechanical ventilation and number of ventilation days
  - Proportion of patients who need ECMO and number of days on ECMO
- Lung function (partial oxygen pressure, fraction of inspired oxygen, Horowitz index)
  - Absolute PaO2 (mmHg) values and change compared to BL
  - Absolute FiO2 (mmHg, fraction of inspired oxygen) and its change to BL
  - Horowitz Index (mmHg) and change to BL
    *(Lung function assessments will only be documented for the study if routinely done)*
- Oxygen saturation and breathing rate
  - Oxygen saturation and breathing rate and change to BL
  - Proportion of patients reaching > 92% oxygen saturation under room air
- Systemic inflammation (changes and normalization in troponin, procalcitonin, CRP, leukocyte count, ferritin, D-dimers, lactate, lactate dehydrogenase, albumin)
  - Concentration of blood parameters and their change to BL
  - Proportion of patients with normalized systemic inflammation (for each inflammation parameter)
- Survival
  - Proportion of patients who survived until day 28
  - Proportion of patients who survived until day 60
- Capillary refill time
  - Proportion of patients with normal and pathological capillary refill time at baseline (as pathological: ≥2 s or normal: <2 s) and change to BL
- Duration of hospital stay
  - Length of hospital stay until day 28 or until day 60
  - Proportion of patients that have been discharged from the hospital until day 28 and day 60
- Drug accountability (e.g. dose, number of administrations)
  - Number of IMP administrations
  - Mean IMP dose per day and mean cumulative IMP dose
- SARS-CoV-2 rRT-PCR ct values and time to negativity (i.e. two consecutive negative PCR-tests as defined by each local laboratory)
  - Time to SARS-CoV-2 RT-PCR negativity in respiratory tract specimen
  - Proportion of patients with a negative SARS-CoV-2 PCR test at day 6, 10, and 28
  - Change (reduction) in SARS-CoV ct values
- Subgroup analysis: Concomitant medication and SARS-CoV-2 vaccination status

We describe the subgroup analyses descriptively for the treatment groups. We also reserve the right to compare them between groups with exploratory statistical tests.

#### Explorative objective

- Capillary leak assessment (plasma ADM)
  - Plasma ADM amount and change to BL
- Blood plasma phenotyping: endothelial dysfunction and immunological characterization
  - Amount of von Willebrand factor (vWF), plasminogen activator inhibitor-1 (PAI-1), ICAM-1, VCAM-1, E-selectin, and change to BL
  - IL-1α, IL-6, IL-10
  - OMICs analysis focusing on immunological parameters
- Quality of life, healthcare use, and productivity
  - EQ5D-5L score (also single questions) at day 28 and FU
  - Use of healthcare resources and productivity assessed at FU

#### Safety

- Safety parameters (evaluated for the safety set)
  - Number of adverse events and serious adverse events
  - Type and severity (mild, moderate, severe) of (serious) adverse events
  - Seriousness and relatedness of (S)AEs
  - Mean number per patient and total number of injection-related (S)AEs within 1 h after injection of IMP
  - Treatment-related (S)AEs

Any clinical adverse event or abnormal laboratory test value that is serious, irrespective of the treatment received by the patient, must be reported to the sponsor by the investigator within 24 h of knowledge. We use the definition and reporting requirements according to local Drug Law, GCP, and ICH Guideline for Clinical Safety Data Management. As survival/death is one endpoint parameter, the integrity of the clinical trial may be compromised when the blind is systematically broken by reporting fatal and/or life-threatening SUSARs. Therefore, fatal and/or life-threatening SAEs that fulfil the following criteria will be treated as disease related and will not be subject to systematic unblinding and expedited reporting:

- Haemodynamic worsening
- Liver cytolysis/insufficiency
- Acute renal failure
- Diffuse intra-vascular coagulation/bleedings
- Leucopenia/thrombopenia/anaemia
- Severe mesenteric ischaemia
- Brain death/haemorragic stroke/ischaemic stroke
- Pulmonary embolism
- Acute myocardial infarction/cardiac rhythm disorders
- Aspiration pneumonia/ventilator-acquired pneumonia/bacteraemia/fungaemia

Adverse events, especially those for which the relationship to test “drug” is “related”, should be followed up until resolved or until FU visit. If a clear explanation is established, it should be recorded on the CRF. Treatment of AEs is at the discretion of the investigator and should follow the standards of medical care at the investigator’s institution.

#### Insurance

Insurance cover is provided for damage to health that occurred no later than 10 years after completion of the clinical trial conducted on the insured person and is reported to the insurer no later than 10 years after completion of the clinical trial

#### Study management groups

IXION is embedded in the EU-founded project COVend. Therefore, infrastructure required for the EU project (e.g. Steering committee and data manager) according to the grand agreement will be used in IXION. To ensure the highest safety level, we established an external scientific advisory board, a risk assessment group, a data safety monitoring board, and an ethical advisory board.

#### Data safety monitoring board

A data safety monitoring board (DSMB) will be established for this study. The DSMB is an independent advisory group of experts with a mandate to periodically review blinded and unblinded safety data to allow ongoing judgment on the benefit and risk of the trial. The DSMB can recommend that the study should be stopped, temporarily suspended, or amended if it determines that the study is not meeting the necessary safety requirements (e.g. new information is available which changes the benefit-risk balance to the negative). The DSMB consists of physicians and scientists with clinical and scientific expertise in clinical studies.

To ensure a high level of data security, a GDPR data protection concept was established for the project. In addition, all participating partners must also have a data protection concept. Further information can be found in the respective data protection concepts.

#### Data access

During the course of the study, the medical study staff will of course have access to the eCRF (with pseudonymized data), as well as the data management, monitors, laboratory staff, PMO, sponsor, and project management (all blinded to the study medication). The Drug Safety Physician as well as the DSMB receive the required safety extracts from the database upon request or prior to a DSMB meeting and have the possibility to unblind the therapy data if necessary. Laboratory results for phenotyping of the blood plasma (ITMP laboratory) are transmitted monthly in CSV format and imported into the study database after conversion into XML format by the data management. The statistics receive the study data for evaluation, and after completion of the study, the sponsor receives the entire pseudonymized data and the investigators receive the data for their respective study centre (pdf files on data carriers). In any case, only pseudonymized data will be passed on.

During the study, only authorized representatives of the sponsor (e.g. CRAs and auditors) and the responsible monitoring authorities have access to personal health data (patient files at the study centre) in addition to the study personnel at the study centre.

After the completion of the study, no further studies are planned with the collected data and biological specimens.

### Sample size considerations

Based on our literature research [24–26], we estimate the frequency of patients who do not worsen their disease status under placebo (+SoC) to be 70%, since we include only hospitalized COVID-19 patients and not any ambulatory COVID-19 patients with mild disease state. We expect that FX06 (+SoC) improves this rate by approximately 15%. Following Spinner et al. and ICU estimations at the KGU Frankfurt, we also expect 15% less patients with progressed/worsened disease status in the group treated with FX06 (+SoC) compared to placebo (+SoC).

Applying a *Z*-test for proportions with continuity correction with a significance level of 5%, *n* = 291 patients (194 FX06: 97 placebo; without drop-outs) are required to demonstrate a treatment difference in the proportion of patients with progressed/worsened disease until day 28 with a power of 80%. Considering 5% drop-out, 306 (204 FX06: 102 placebo) patients need to be recruited.

Sample size was calculated with G*Power version 3.1.9.6 using the *Z*-test for the difference between two independent proportions with continuity correction. Sample sizes were reconfirmed using PASS 16.0.2.

### Statistical methods

The statistical analysis will be performed by the study statistician after termination of the study, when all included patients have finished their last visit, the data review process of the data management is completed, and the database is hard locked and when all data queries are resolved. Data will be unblinded after the database has been hard locked. The following presentation is a short summary of applied methods. A detailed description will be given in the provided additional files.

### Definition of population for analysis

The study includes eligible hospitalized non-intubated COVID-19 patients according to inclusion and exclusion criteria.

#### All randomized included patients (RS, randomized set)

The randomized set (RS) is defined to include all participating patients who were randomized into the study. Note that drop-outs of randomized patients will only occur if the patient does not take at least one dose of medication (these are not part of the analysis sets other than RS) and/or if the patient withdraws consent and/or if the investigator/PMO/sponsor withdraws the patient from the study. If a patient is withdrawn from the IMP, they are not withdrawn from study and therefore remain part of the analyses sets.

#### Full analysis set (FAS)

For the full analysis set (FAS) for the final analysis of the primary and secondary objectives, we will only consider the subgroup of all randomized included patients (RS) who had a baseline and one post-baseline assessment of their disease state based on the WHO score and who received at least one dose of FX06/placebo during the study. Otherwise, the considered primary endpoint and thus, the treatment effect, cannot be estimated under IMP use. Note that the FAS may be equal to the RS.

#### Per-protocol population

Per protocol population (PP) is defined as the subset of the FAS excluding those patients with major protocol violations occurring up to day 28. Specific reasons for warranting exclusion will be documented prior to the closing of the database. Not all protocol deviators and violators will be excluded from the per protocol population.

#### Safety-population

The safety analysis set is defined as all randomized patients who received at least one dose of FX06/placebo during the study. This population is a subset of all randomized included patients (RS) and will be used for all safety analyses.

#### Drop-outs

Drop-outs of randomized patients will only occur if the patient does not take at least one dose of medication (these are not part of the analysis sets other than RS) and/or if the patient withdraws consent and/or if the investigator/PMO/sponsor withdraws the patient from the study.

If a patient is withdrawn from the IMP, they are not withdrawn from study and therefore remain part of the analysis sets.

We expect a high intrinsic motivation from the patients. IXION is a medical class II study, conducted by professional study centres under monitor supervision. Furthermore, the study burdens for the patient are kept as low as possible, e.g. interim visits can be done remotely.

#### Missing values

We do not expect many regular missing values due to the close visit windows and patient care. Collection of the WHO score up to day 28 will be tightly controlled for. In general, real missing data of a parameter will not be transformed and remains untouched unless stated otherwise. Summary statistics will be given including the underlying number of valid individual values and missing visit-wise.

We predefine the following measures for handling data of selected analyses:

All patients who die prior to day 28 will be given the corresponding highest WHO score (score of 10) for all remaining visits at and after assessment visit of their death for the purpose of assessing the primary endpoint and related sensitivity analyses even though no further patient data can be collected after visit of death. The date of death will be considered the time point for the time-to-event analyses. In case of drop-outs prior to day 28 or missing WHO scores of the FAS population prior to day 28 it will be evaluated, if a statistical deduction of the primary endpoint is possible independent of missing WHO scores, e.g. because the patient shows a worsened WHO score at some point, or no more than 3 consecutive visits show a worsened WHO score. If such a deduction of the primary endpoint is not possible, a decision on missing WHO scores may be deduced by taking a closer look at these cases at the blind data review meeting where all available information on the patient is evaluated in a blinded manner. All remaining patients of the FAS where no WHO score can be assigned will be considered missing for the primary analyses and will be considered by different scenarios as sensitivity analyses of the primary analysis (best/worse-case scenario and by LOCF). For all further endpoints, no imputation is planned currently.

Note that linear mixed models using maximum likelihood estimation are able to deal with missing values and are a valid alternative to imputation. As such, laboratory assessments are expected to be modelled accurately in terms of missing in LMMs.

Unless stated otherwise, values below/above the LOQ will in general be imputed by a suitable fixed value (e.g. LLOQ, ULOQ, Zero), depending on the nature of the laboratory value and its time of assessment. If a large number of measurements of a laboratory parameter falls above/below the LOQ, alternative methods will be considered. SARS-CoV-2 antibodies below the threshold (LOQ) will be defined as 0/not present for the purpose of analyses.

#### Statistical analyses

As primary analysis, we will compare the difference in proportion of patients with progressed/worsened disease state (as defined according to WHO score) until day 28 in both treatment groups (2-sample chi squared test). The aim is to demonstrate preliminary superiority of FX06 in prevention of worsened disease state in comparison to placebo (with standard of care) treatment. For the group comparison, the 5% significance level is considered.

With regard to secondary endpoints, these will be described by summary statistics stratified by treatment group and available visits and the treatment difference until day 28; the 95% confidence interval will be given. For selected secondary endpoints, linear mixed models will be applied to investigate the impact of treatment, time, treatment-by-time, and other factors such as demographics.

## Discussion

### Bias and limitations

We expect a neglectable bias with regard to lack of compliance since all patients will be treated in hospital the entire time during the treatment period. Since we investigate the disease progression under FX06 treatment in several countries, a bias might arise when quality standard of care differs between countries. While this might impact the size of the primary endpoint, the assumptions on the treatment difference of the primary endpoint should not be impacted, since the randomization protects from imbalances within this regard within the different countries. Furthermore, as sensitivity analyses, endpoints will be compared descriptively among centres, or as effects of the regression models. Due to the double-blind study design, the risk of potential biases should be minimized.

Potential limitations might refer to a lack of recruiting and drop-out due to various possible protocol violations. While we controlled for drop-outs in the same size estimation, recruitment problems may be subject to external problems difficult to control for. One reason for the difficult recruitment are the competing ongoing trials on COVID-19, while at the same time a patient may not want to be assigned a placebo treatment although it represents standard of care.

### Discussion

The ongoing COVID-19 pandemic is affecting people worldwide since the first reported case. Up to the end of March 2022, more than 460 million cases and up to 6 million deaths have been reported [27].

With changing variants and the anti-vaccine movement, COVID-19 is still putting stress on ICUs. Therefore, we need new and diverse pharmaceutical options to treat COVID-19. FX06 might be a promising approach by targeting the inflamed endothelium of the host.

The IXION trial will answer the question whether FX06 has the potential to prevent progression from a moderate to severe/critical COVID-19 stage.

Furthermore, we like to identify specific molecular “Fingerprints” of the complex interaction between the endothelium and immune system. This might help to understand other conditions with endothelial dysfunction and capillary leak (e.g. sepsis and acute respiratory distress syndrome).

### Trial status

Study protocol (V1.1) was finalized at 12th of January 2022. This trial was registered with the responsible federal authority (Bundesinstitut für Arzneimittel und Medizinprodukte – No. 61-3910-4045190) and European Union Drug Regulating Authorities Clinical Trials Database (EudraCT no.: 2021-005059-35). Recruitment and enrolment of the patients will start at the end of 2022. The planned recruitment lasts 18 months.

## Supplementary Information

**Additional file 1.** Additional Information – Further statistical Information for IXION.

## Data Availability

The datasets generated and/or analysed during the current study are available from the corresponding author on reasonable request after the end of the trial.

## Abbreviations

ACE2: Angiotensin-converting enzyme 2
AE: Adverse event
ALT: Alanine aminotransferase
ARDS: Acute respiratory distress syndrome
AST: Aspartate aminotransferase
bio-ADM: Bioactive Adrenomedullin
BL: Baseline
BMI: Body mass index
C: Concentration
CI: Confidence interval
Cl: Clearance
COPD: Chronic obstructive pulmonary disease
COVID-19: Coronavirus disease 2019
CRP: C-reactive protein
CRT: Capillary refill time
ct: Cycle threshold
DMP: Data Management Plan
e.g.: Exempli gratia; for example
ECMO: Extracorporeal membrane oxygenation
FiO2: Fraction of inspired oxygen
FU: Follow-up
GCP: Good Clinical Practice
GCSF: Granulocyte-colony stimulating factor
GGT: Gamma-glutamyl transferase
GMP: Good Medical Practice
HI: Horowitz index
i.v./IV: Intravenous
(S)AEs: (Serious) adverse event
ICAM-1: Intercellular adhesion molecule 1
EQ-5D-5L: Patient Reported Outcome (PRO)-Instrument
ICU: Intensive care unit
IgG: Immunoglobulin G
IIT: Investigator-initiated trial
IL: Interleukin
IMP: Investigational Medicinal Product
IUD: Intrauterine devices
MRI: Magnetic resonance imaging
SoC: Standard of care
SCR: Screening
STEMI: ST-segment elevation myocardial infarction
WHO: World Health Organization
