# Supplementary material for: Potential of FX06 to prevent disease progression in hospitalized non-intubated COVID-19 patients — the randomized, EU-wide, placebo-controlled, phase II study design of IXION
Source: Trials. 2022 Aug 19;23:688. doi: 10.1186/s13063-022-06609-x (PMC9389510; doi:10.1186/s13063-022-06609-x)
Supplement: Supplementary file 1 — Additional file 1. Additional Information – Further statistical Information for IXION. [file 13063_2022_6609_MOESM1_ESM.docx]

**Additional Information – Further statistical Information for IXION**

**Randomization**

A randomization list will be generated by an authorized person through the Sponsor. Central randomization will be performed in a 2:1 allocation to FX06 and placebo stratified by WHO group (moderate: score 4-5 and severe: score 6) and centre. As randomization procedure, stratified block randomization will be applied. The randomization will be performed centrally within the eCRF System Open Clinica.

Independent pharmacists will dispense the allocated treatment according to the computer-generated randomization list.

**Allocation**

The randomization list is not available to blinded study staff including treating physicians. Within the clinics, treatment allocation will be concealed by providing placebo IMP for FX06, which will be of identical appearance in comparison to verum.

**Implementation**

The randomization sequence is generated and validated prior to study start by the data management. Eligible patients are enrolled by the treating investigator who allocates the patients to the next randomized treatment according to the computer generated randomization list. Blinded IMP is then given according to randomization number.

**Blinding**

The study is randomized and double-blinded for patients and physicians (outcome assessors). Treatment allocation remains concealed throughout the study, minimizing the risk of assessment and selection bias and assessment bias.

The treatment allocation also remains blinded for the study statistician until the final analysis to avoid early data driven conclusions.

The blinding is intended to limit the occurrence of conscious and unconscious bias in the conduct and interpretation of a clinical trial arising from the influence that the knowledge of treatment may have on the recruitment and allocation of subjects to treatments, their subsequent care, the attitudes of subjects to the treatments, the assessment of endpoints, the handling of withdrawals, and so on.

**Sample size considerations**

Gottlieb et al (2021) compared the effects of Bamlanivimab to placebo in 577 ambulatory patients with mild to moderate COVID-19. The frequency of COVID-related hospitalizations or ER events at day 29 in high-risk patients were 2.7-5.9% for Bamlanivimab (700 mg – 7000 mg), 0% (Bamlanivimab+etesevimab) and 13.5% for placebo^1^. Similarly, Spinner et al (2020), found that remdesivir led to 5% to 10% more clinical improvement than placebo /Standard of Care (REM :70%/65% vs PL :61%) after 7 to 11 days in 585 hospitalized COVID-19 patients with moderate to severe disease status^2^. Currently no efficacy data is available on FX06 treated COVID-19 patients, thus, deductions can be drawn from other therapies.

Based on our literature research^3^ ^1, 2, 4^, we estimate the frequency of patients who do not worsen their disease status under Placebo (+SoC) to be 70%, since we include only hospitalized COVID-19 patients and not any ambulatory COVID-19 patients with mild disease state. We expect that FX06 (+SoC) improves this rate by approximately 15%. Following Spinner et al and ICU estimations at the KGU Frankfurt, we also expect 15% less patients with progressed/worsened disease status in the group treated with FX06 (+SoC) compared to placebo (+SoC). Any improvement is evaluated as clinically relevant. A 2:1 (FX06: PL) allocation is used, since we expect the treatment to be beneficial. Drop-outs are considered to be low (5-10%) since death and efficacy related changes are accounted for by the endpoint. We assume an expected treatment difference in proportion of patients with progressed/worsened disease defined according to WHO scale until day 28 to be 15% (15% for FX06, 30% for placebo). Null hypothesis considers equal probabilities for the primary endpoint (p) in both treatment groups (H0: δ = p_FX06_ – p_PL_ = 0 vs. H1: δ ≠ 0, in favour of FX06 treatment, i.e. p_FX06_ < p_PL_).

Applying a Z-test for proportions with continuity correction with a significance level of 5%, n=291 patients (194 FX06: 97 placebo; without drop-outs) are required to demonstrate a treatment difference in the proportion of patients with progressed/worsened disease until day 28 with a power of 80%. Considering 5% drop-out, 306 (204 FX06: 102 placebo) patients need to be recruited.

Sample size was calculated with G*Power Version 3.1.9.6 using the Z-test for the difference between two independent proportions with continuity correction. Sample sizes were reconfirmed using PASS 16.0.2.

**Statistical Methods**

The statistical analysis will be performed by the study statistician after termination of the study, when all included patients have finished their last visit, the data review process of the data management is completed, and the database is hard locked and when all data queries are resolved. Data will be unblinded after the database has been hard locked. The following presentation is a short summary of applied methods. A detailed description will be given in a statistical analyses plan.

Analyses sets:

The randomized set (RS) is defined to include all participating patients who were randomized into the study. Furthermore, we define the full analysis set FAS for the final analysis of the primary and secondary objectives as the subgroup of all randomized included patients (RS) who had a baseline and one post-baseline assessment of their disease state based on the WHO score, and who received at least one dose of FX06/placebo during the study. Otherwise, the considered primary endpoint and thus, the treatment effect, cannot be estimated under IMP use.

Furthermore, the per protocol set PP is defined as the FAS excluding those patients with major protocol violations occurring up to day 28. And the safety set is defined as all randomized patients who received at least one dose of FX06/placebo during the study.

Drop-outs:

Drop-outs of randomized patients will only occur if the patient does not take at least one dose of medication (these are not part of the analyses sets other than RS) and/or if the patient withdraws consent and/or if the investigator/PMO/sponsor withdraws the patient from the study.

If a patient is withdrawn from the IMP they are not withdrawn from study and therefore remain part of the analyses sets.

Missing Values:

We do not expect many regular missing values due to the close visit windows and patient care. Collection of the WHO Score up to day 28 will be tightly controlled for. All patients who die prior to day 28 will be given the corresponding highest WHO score (score of 10) for all remaining visits at and after assessment visit of their death for the purpose of assessing the primary endpoint.

In case of drop outs prior to day 28 or missing WHO scores of the FAS population prior to day 28 it will be evaluated, if a deduction of the primary endpoint can be derived statistically or from the individual patient data in the blinded data review meeting. Otherwise, these patients are only considered for sensitivity analyses of the primary analyses by best/worst case scenarios. For all further endpoints, no imputation is planned currently. Note that linear mixed models using maximum likelihood estimation are able to deal with missing values and are a valid alternative to imputation.

Values below/above the LOQ will in general be imputed by a suitable fixed value (e.g., LLOQ, ULOQ, Zero), depending on the nature of the laboratory value and its time of assessment.

Note that imputation methods may be reconsidered based on distributional behaviour.

Statistical Analyses:

As primary analysis we will compare the difference in proportion of patients with progressed/worsened disease state (as defined according to WHO score) until day 28 in both treatment groups (2-sample chi squared test for independent proportions, 2-sided design at α=5%. The aim is to demonstrate preliminary superiority of FX06 in prevention of worsened disease state in comparison to placebo (with standard of care) treatment. Primary analysis will be performed as-randomized for the FAS and the PP set, including randomized patients withdrawn from treatment. With regard to estimands, we follow the treatment-policy in general, if data points are missing, both composite strategy is applied and a sensitivity analysis is performed using hypothetical strategy: Patients of the FAS with missing primary endpoint due to missing WHO scores, will be considered missing for the primary analyses and will be considered by different scenarios as sensitivity analyses of the primary analysis (best/worse case scenario and by LOCF). As a further sensitivity analyses a generalized linear mixed model (logit) is fit to evaluate the impact of the strata (random effects) on the odds ratio of a worsened WHO state between the treatment groups (further covariates such as age, gender and preconditions may be considered). For the group comparison the 5% significance level is considered.

With regard to secondary endpoints, these will be described by summary statistics stratified by treatment group and available visits, and the treatment difference until day 28 the 95% confidence interval will be given. For selected secondary endpoints (e.g. WHO score, lung function, blood parameters of systemic inflammation) linear mixed models will be applied to investigate the impact of treatment, time, treatment-by-time and other factors such as demographics.

Safety data will be investigated descriptively by summary statistics. Explorative statistical tests will be applied if suitable. Time-to-event analysis may be performed to compare time-to-AE between treatments for frequent adverse events.

Explorative treatment and subgroup comparisons of all endpoints may be performed by appropriate statistical tests at an explorative 2-sided 5% / or 1-sided 2.5% significance level (e.g. Chi Squared, Fisher test, Wilcoxon tests, van Elteren tests, CMH tests, t-/F-Tests, log rank tests). An adequate multiple testing procedure (e.g. Benjamin Hochberg, Bonferroni-Holm or Tukey), will be considered in case of multiplicity. In that case, all adjusted and unadjusted p-values will be reported.

Since data is collected from several centres in different countries, a centre and country effect may arise. To investigate the homogeneity between the centres and the countries, the primary, selected secondary and safety endpoints will be compared exploratively between centres and countries using summary statistics stratified by treatment. Alternatively, centres/country effects will be incorporated in corresponding statistical models.

**References**

1. Gottlieb RL, Nirula A, Chen P, Boscia J, Heller B, Morris J, et al. Effect of Bamlanivimab as Monotherapy or in Combination With Etesevimab on Viral Load in Patients With Mild to Moderate COVID-19: A Randomized Clinical Trial. JAMA. 2021;325(7):632-44.

2. Spinner CD, Gottlieb RL, Criner GJ, Arribas Lopez JR, Cattelan AM, Soriano Viladomiu A, et al. Effect of Remdesivir vs Standard Care on Clinical Status at 11 Days in Patients With Moderate COVID-19: A Randomized Clinical Trial. JAMA. 2020;324(11):1048-57.

3. Guan WJ, Ni ZY, Hu Y, Liang WH, Ou CQ, He JX, et al. Clinical Characteristics of Coronavirus Disease 2019 in China. N Engl J Med. 2020;382(18):1708-20.

4. Huang C, Wang Y, Li X, Ren L, Zhao J, Hu Y, et al. Clinical features of patients infected with 2019 novel coronavirus in Wuhan, China. Lancet. 2020;395(10223):497-506.
